# Social Support and Health in Diabetes Patients: An Observational Study in Six European Countries in an Era of Austerity

**DOI:** 10.1371/journal.pone.0135079

**Published:** 2015-08-25

**Authors:** Jan Koetsenruijter, Jan van Lieshout, Christos Lionis, Maria Carmen Portillo, Ivo Vassilev, Elka Todorova, Christina Foss, Manuel Serrano Gil, Ingrid Ruud Knutsen, Agapi Angelaki, Agurtzane Mujika, Poli Roukova, Anne Kennedy, Anne Rogers, Michel Wensing

**Affiliations:** 1 Radboud university medical center, Radboud Institute for Health Sciences, Department of IQ healthcare, Nijmegen, The Netherlands; 2 Clinic of Social and Family Medicine, Faculty of Medicine, University of Crete, Heraklion, Greece; 3 School of Nursing, University of Navarra, Pamplona, Spain; 4 NIHR Wessex CLAHRC, Faculty of Health Sciences, University of Southampton, Hampshire, United Kingdom; 5 Department of Economic Sociology, University of National and World Economy, Sofia, Bulgaria; 6 University of Oslo, Institute for Health and Society, Oslo, Norway; 7 Education, Health and Society Foundation, Murcia, Spain; Örebro University, SWEDEN

## Abstract

**Introduction:**

Support from individual social networks, community organizations and neighborhoods is associated with better self-management and health outcomes. This international study examined the relative impact of different types of support on health and health-related behaviors in patients with type 2 diabetes.

**Methods:**

Observational study (using interviews and questionnaires) in a sample of 1,692 type 2 diabetes patients with 5,433 connections from Bulgaria, Greece, Netherlands, Norway, Spain, and the United Kingdom. Outcomes were patient-reported health status (SF-12), physical exercise (RAPA), diet and smoking (SDCSCA). Random coefficient regression models were used to examine linkages with individual networks, community organizations, and neighborhood type (deprived rural, deprived urban, or affluent urban).

**Results:**

Patients had a median of 3 support connections and 34.6% participated in community organizations. Controlled for patients’ age, sex, education, income and comorbidities, large emotional support networks were associated with decrease of non-smoking (OR = 0.87). Large practical support networks were associated with worse physical and mental health (B = -0.46 and -0.27 respectively) and less physical activity (OR = 0.90). Participation in community organizations was associated with better physical and mental health (B = 1.39 and 1.22, respectively) and, in patients with low income, with more physical activity (OR = 1.53).

**Discussion:**

Participation in community organizations was most consistently related to better health status. Many diabetes patients have individual support networks, but this study did not provide evidence to increase their size as a public health strategy. The consistent association between participation in community organizations and health status provides a clear target for interventions and policies.

## Introduction

Self-management is a key component of the management of many long-term conditions, including type 2 diabetes. Ageing populations and unhealthy lifestyles are attributed with responsibility for an increase in the prevalence of these conditions. For example, in the European Union, about 53 million adults aged 20–79 years had diabetes in 2013 and this is predicted to increase to 64 million in 2030 [1]. As part of recent austerity measures in recent years, public services have increasingly made chronic-disease patients responsible for the management of their own health. This transfer of responsibility has greatest impact on patients with low incomes, as austerity measures affect them more than affluent groups and they also have the capacity to benefit most from self-management support [2–4]. These developments have increased the burden on individual patients with long term conditions and raise the question whether all patients receive the support they need to manage their health and diseases.

Although self-management has often been defined in terms of individual competencies, its effectiveness is increasingly perceived to be influenced by social support [5–7]. Social support for patients with chronic diseases is help provided by family, friends, neighbors or others; it includes different domains, such as information, emotional comfort, and practical help. This support is provided through individual social networks with family and friends, but also in community organizations and local neighborhoods. Social support may function as a compensation for austerity measures as previous research found that large and varied individual social networks were associated with better health outcomes [8,9]. Different mechanisms relating to how networks can affect health have been identified. Individual networks can help the patient to navigate individuals to available sources of support and influence the coordination of support activities [10,11]. Contagion of ideas and behaviors has been suggested to explain the impact of being embedded in a group or population, such as a community organization [12]. Also, neighborhoods can influence population health by their physical and social lay out [13].

Within individual countries, some indication is given how the social context can contribute to better health. A study in the UK suggests that community and network-centered approaches may be particularly relevant for engaging people in socially and economically deprived areas [7]. Another study in the United Kingdom explored social support systems of people with diabetes [14]. In Norway, poor social integration among elderly was related to higher mortality [15], and in the Netherlands poor emotional support was related to higher mortality [16]. In Spain a study found that among elderly a low social network was related to more hospital admissions [17] and that social support offered protection against the adverse effect of economic recessions on mental health [18]. However, most of these studies focused on single factors and single settings and therefore it is unclear what the relative impact of different aspects of support on health is.

In this international study, we aimed to describe the social support available to patients with type 2 diabetes and to identify which aspects of social support are related to health and health-related behaviors in patients with type 2 diabetes in a variety of European countries. Moreover, we investigated whether these relationships differ between high and low income groups, in order to explore whether social support can compensate for the adverse health effects of deprivation and austerity.

## Methods

### Study design, setting and participants

We conducted an international cross-sectional study in patients with type 2 diabetes. Data were collected as part of the EU-WISE project which is an European project based on the WISE (Whole System Informing Self-management Engagement) approach and was funded by the European Union Seventh Framework Programme (FP7) Health [19,20]. The study was conducted in 18 purposefully chosen geographic areas in 6 countries, which reflect a variety of health and welfare systems: Bulgaria, Greece, the Netherlands, Norway, Spain, and the UK. Patients were recruited through healthcare practices. Each of the participating countries selected a deprived urban area; a relatively affluent urban area; and a deprived rural area (relative to country). We defined urban as located in a city with more than 100,000 inhabitants and rural as located in towns or villages with fewer than 30,000 inhabitants. This selection of areas (rather than a random sample) allowed us to study both individual and area characteristics. Because the areas were chosen purposefully and not randomly, the areas are not necessarily representative for the countries involved. In each area, 100 patients with a medical diagnosis of type 2 diabetes were recruited resulting in about 300 patients in each country. This number allowed us to detect a medium effect size (*f*2 = 0.15) based on α = 0.05, intraclass correlation coefficient (ICC) = 0.03, power = 0.80 and the inclusion of eight independent variables in the analysis [19,21]. Inclusion criteria were: medical diagnosis of diabetes; type 2 diabetes only; age of 18 years or over. Exclusion criteria were: pregnancy; pregnancy-related diabetes; recent/current major surgery or medical procedures; severe cognitive or psychiatric handicap; terminal illness/receiving palliative care; absence of translators (e.g. family members) for patients with insufficient language skills. Eligible patients were given an invitation letter with information, a consent form, and a written questionnaire via their healthcare practice. Patients who completed the questionnaire were invited to participate in an interview as well. Written informed consent was given by all patients. Ethical committees in the different countries provided approval for the study; The UNWE and the NCPHA (National Center for Public Health and Analysis) in Bulgaria, the Scientific & Bio-ethical Committee and the Administration Council of the Regional Academic Hospital (PAGNI) of Heraklion in Greece, the CMO region Arnhem Nijmegen in The Netherlands, the Regional Committee for Health and Research Ethics and the ethical committee of the Oslo University Hospital, the Ethics Commission of the University of Navarra, and the University of Manchester Research Ethics Committee, the Greater Manchester Research Ethics Committee, Salford and Trafford local research ethics committee, and the University of Southampton Ethics and Research Governance Online in the UK.

### Measures

The study used a pre-structured patient questionnaire, which consisted of two parts: first a written questionnaire with validated measures recording demographic variables, quality of life, self-care behaviors, received care and participation in local organizations. The second part was a face-to-face or telephone interview, which focused on social networks and social support. The choice of interviews was based on pilot testing which suggested that written surveys of the measures were not feasible in the targeted population.

#### Social support measures: individual support networks and community organizations

Data on numbers of household members, presence of spouses and participation in community organizations were gathered using structured questions (3 items). Attending community organizations was based on the question which community groups, activities or services the respondent visited in the last 6 months. Examples of these groups could involve: well-being, internet communities, health education, practical support, healthy eating, physical activity or transport. Data on individual support networks was collected through interviews using a validated name generator method [22]. This method first requires a respondent to name actual persons and then several additional questions about these individuals were asked. For each individual mentioned through the name generator, the following 9 items were derived: gender, age, type of relationship, duration of relationship, distance to member, and, the number of members that provided information, practical or emotional support. Information was defined as information related to dealing with someone’s illness; practical support as receiving help with practical things in and around the house; emotional support was defined as talking about health problems or other personal problems. Finally, the position generator was used to identify access to people with specified healthcare professions (nurse, doctor or pharmacist) [23]. This method measures access to network members' occupations that functions as a source for social capital [24]. These people were not necessarily part of the patient’s self-reported support network, as they could be in the patient’s wider environment, defined as all their friends and family members.

#### Other measures

We measured income relative to the country’s average income. Respondents answered whether their income was below/about/above the country yearly average income (BG 4,500 lev; GR 12,000 EUR; NL 33,000 EUR; NO 350,000 NKr.; ES 22,800 EUR; UK 25,000 pound). We defined low income as those whose income was below the country yearly average income. We regarded someone as non-native if one of the parents was born in a different country. In addition, a short list of 9 comorbidities was included.

#### Outcomes: health and health-related behaviors

We used the SF-12 (version 2) to measure functional health status, both a physical and mental component [25]. The SF-12 was devised as a shortened version of the SF-36 which is a set of generic, coherent, and easily self-administered health related quality-of-life measures, developed by RAND as part of the Medical Outcomes Study (MOS) [26]. The physical (PCS) and mental (MCS) component are subsets of the SF-12. To assess self-management behavior we used 3 indicators: physical activity, healthy diet and non-smoking. These indicators were measured by 2 validated scales: the Summary of Diabetes Self-Care Activities (SDSCA) and the Rapid Assessment of Physical Activity (RAPA). The SDSCA assesses self-care behavior and lifestyle, including diet, smoking, physical exercise, blood sugar testing and foot care [27]. The SDSCA was used for diet (2 items measuring general diet) and smoking. Non-smoking was defined as not having smoked a single cigarette in the preceding seven days and a healthy diet was defined as following a healthy eating plan for at least six days a week. The RAPA is used for a more detailed measurement of physical lifestyle of respondents. This questionnaire was specifically developed to measure the level of physical activity of older patients [28]. Healthy physical activity was defined as; doing moderate physical activities at least 30 minutes a day, 5 or more days a week or; doing vigorous physical activities 20 minutes a day, 3 or more days a week. Although all measures were chosen based on proven validity and reliability and often already were translated into other languages, not all measures were available in all countries. Measures that were not yet available in all countries were translated into the specific language using forward- and back-translation and were culturally adapted to the specific country characteristics. In Bulgaria, the SCSCA, RAPA, and, SF-12 were translated independently by two researchers into the Bulgarian language. Consensus on the both translations was done by a third researcher and the final version of the translated questionnaire was translated back by a professional translator. The procedure for the heiQ translation followed the same procedure, and also included an extensive discussion with the developer of the heiQ (Richard Osborn, Deakin University), so that the Bulgarian team and UK team received an official license to use in Bulgaria. In Greece, the RAPA was translated by three researchers and a professional translator independently. After reaching consensus, back translation was done by the same team and cultural adaptations were made. The newly translated questionnaire was pilot tested in 3 diabetes type 2 patients in order to test clarity and understanding.

### Statistical analysis

We calculated descriptives per country of patient characteristics with measures for individual characteristics and social support characteristics. The same descriptives were presented for the measures of self-management (physical activity, diet and smoking) and physical and mental health status.

To determine the effect of social support on self-management we performed a regression analysis with self-management and health status as dependent variables and individual and social support characteristics as independent variables. Physical and mental health status were treated as interval scale and therefore analyzed using an ordinary least squares (OLS) linear regression model; physical activity, diet and smoking were dichotomized and therefore analyzed using a logistic regression model. The regression analyses were based on a multilevel model with a random slope with patients nested within areas and both nested in the six participating countries [29]. All analyses were controlled for patients’ age, gender, comorbidity and educational background as potential confounders.

We first tested the effects of income and social support for the whole sample. Secondly, we performed the same analysis separately for high and low income groups, to identify if the effects (e.g. of social networks on self-management behaviors) were different between level of deprivation. All regression analyses were performed for each country separately to check for patterns within countries. Countries and areas were not randomly sampled, and therefore both levels were considered fixed factors implying that generalization beyond chosen areas and countries was avoided. Significance was indicated by p <0.05 and analyses were performed using IBM SPSS statistics 20 (IBM Corp.) and MLwiN 2.28 (Centre for Multilevel Modelling).

## Results

In total 1,861 patients completed the written questionnaire, of which 1,692 also participated in the interview. Average age was 66.2 year, men and women were equally present (50.0% female) and 6.2% had a non-native origin. The majority (61.0%) had an income that was below the country average, which reflects the focus of the study on deprived populations. They reported 5,433 connections with individuals providing some kind of support (a mean of 3.2 connections per patient and a median of 3 connections per patient). Nearly half (48.3%) had health professionals in their extended network and about a third (34.6%) participated in community organizations. Regarding self-management behavior, a physically-active lifestyle was reported by 35.3%, 50.8% followed a healthy diet and 85.8% were non-smokers (Table 1).

**Table 1:** Description of patient samples.

	*Total (n = 1692)*	*Bulgaria (n = 283)*	*Greece (n = 302)*	*Netherlands (n = 245)*	*Norway (n = 291)*	*Spain (n = 290)*	*UK (n = 281)*
**Individual characteristics**							
*Sex (% female)*	50.0	61.1	57.3	43.8	38.5	55.9	40.0
*Age in years (mean)*	66.2	65.2	69.0	68.4	59.8	69.3	65.5
*Parents born in other country (%)*	6.3	0.4	8.6	13.9	14.4	1.0	- †
*Pet in household (% yes)*	38.0	55.0	53.5	29.1	30.7	30.5	27.3
*Education (mean years)*	10.3	10.5	7.8	11.0	11.1	9.0	12.7
*Pensioner (%)*	62.3	70.9	72.7	60.6	29.7	75.3	64.4
*Low income*	61.0	69.3	55.5	47.5	46.2	81.1	65.6
*Comorbidities*							
*0 comorbidities*	14.8	7.4	7.6	13.5	15.1	19.3	26.3
*1–2 Comorbidities*	57.4	52.7	60.6	61.2	51.2	59.7	59.4
*> 2 Comorbidities*	27.8	39.9	31.8	25.3	33.7	21.0	14.2
*Physician/nurse visits last 6 months*							
*0–2 visits*	35.0	14.5	1.0	67.9	39.7	40.9	53.9
*3–5 visits*	51.4	56.0	93.7	29.2	45.2	43.0	34.9
*> 5 visits*	13.5	29.4	5.3	2.9	15.2	16.1	11.2
**Social support**							
*Spouse (% yes)*	70.5	62.1	70.9	74.7	65.6	81.2	71.0
*Household members (mean)*	2.3	2.6	2.3	1.9	2.0	2.6	2.0
*Network members (mean)*	3.2	2.7	2.2	4.1	3.3	3.0	4.1
*Network members providing*:							
*Information support*	1.9	1.9	0.9	1.6	1.9	2.2	2.8
*Practical support*	1.5	1.8	1.2	1.2	1.2	1.6	1.8
*Emotional support*	2.5	2.7	2.1	2.5	2.6	2.6	2.8
*Health professional in wider network (%)*	48.3	54.8	57.6	47.8	49.8	31.4	48.0
*Attending community organizations (%)*	34.6	37.8	24.8	44.1	23.7	41.4	38.1
*Residential area*							
*Urban deprived*	35.9	35.3	32.8	37.6	35.4	33.1	42.3
*Urban affluent*	39.1	32.2	32.8	21.6	30.6	32.8	57.7
*Rural deprived*	25.0	32.5	34.4	40.8	34.0	34.1	-*
**Health and health related behaviors**							
*Health related QOL (SF-12)*							
*Physical (mean)*	50.0	48.0	48.0	51.3	51.8	49.9	50.5
*Mental (mean)*	50.0	48.1	46.9	52.5	51.0	50.7	50.7
*Self-management*							
*Physically active (%)*	35.3	40.1	19.5	49.8	42.9	30.9	31.2
*Healthy diet (%)*	50.8	33.1	56.8	53.1	45.0	67.2	48.9
*Non-smoking (%)*	85.8	80.4	89.0	89.8	77.2	89.2	89.5

*Not included in sampling,

^†^ Not recorded.

Controlled for other patient characteristics and comorbidities, lower income was related to worse physical and mental health status (B = -1.87 and -1.38, respectively) (Table 2). Individual network characteristics were inconsistently related to physical quality of life. Having a spouse was associated with a better physical health status (B = 1.01), especially for patients with a high income, whereas receiving more practical support was associated with a worse physical health status (B = -0.46). Controlled for patients characteristics and comorbidities, attending community organizations was related to better physical health status (B = 1.39). Similar linkages were found for mental health status; having a spouse and visiting community organizations were related to better health status and practical support to worse health status (B = 0.88, 1.22 and -0.27, respectively). In addition, having more health professionals in the wider network was associated with better mental health status (B = 0.67), mostly for patients with a low income (B = 0.76). Patients living in an urban deprived area had worse mental health status compared to patients living in an urban affluent area (B = -0.84), but this effect was only present for patients with a low income (B = -1.29 versus B = -0.11 for high incomes). Effects per individual country are provided in S1 File.

**Table 2:** Linear regression estimates (B) for the relation between social support and health status.

	Physical health related QOL (SF-12)			Mental health related QOL (SF-12)		
	Overall multi-variate	Low income group	High income group	Overall multi-variate	Low income group	High income group
*Age (10 year steps)*	-0.44**	-0.18	-0.86**	0.43**	0.61**	0.18
*Sex (male ref*.*)*	-1.02**	-1.20**	-0.80	-1.17**	-1.33**	-1.11**
*Education*	0.14**	0.16**	0.14*	0.13**	0.16**	0.09*
*Non-native background*	0.02	0.04	0.21	-0.72	-1.18	-0.19
*No comorbidities (ref*.*)*						
*1–2 Comorbidities*	-2.30**	-1.82**	-3.04**	-1.26**	-0.94	-1.77**
*> 2 Comorbidities*	-4.87**	-4.72**	-4.97**	-3.40**	-3.38**	-3.28**
*Low income*	-1.87**			-1.38**		
**Social support**						
*Spouse*	1.01**	0.76	2.45**	0.88**	0.48	1.95
*Household members*	-0.09	-0.04	-0.44	0.14	0.16	0.02
*Support network members (N)*	0.01	0.16	-0.02	0.10	0.26	-0.14
*Network members providing*:						
*Information support*	0.03	0.04	0.00	0.12	0.10	0.21
*Practical support*	-0.46**	-0.36*	-0.70**	-0.27*	-0.25	-0.39*
*Emotional support*	0.13	0.04	0.14	-0.05	-0.08	0.05
*Health professional in wider network*	0.47	0.56	0.28	0.67*	0.76*	0.43
*Attending community organizations*	1.39**	1.32**	1.63**	1.22**	1.38**	1.06*
*Neighborhood (urban affluent = ref*.*)*						
*Urban deprived*	-0.51	-0.49	-0.38	-0.84**	-1.29**	-0.11
*Rural deprived*	0.17	0.36	0.83	0.08	-0.09	0.69

* p <0.05

** p <0.01

Table 3 presents the analysis of health-related lifestyles. Lower income was related to less physical activity (OR = 0.75), but not to diet and smoking (Table 3). Regarding individual network characteristics higher number of practical support connections was associated with less physical activity. However, focusing on differences in level of income, the negative relationship between practical support connections and physical activity only applied to higher incomes (OR = 0.72), whereas for lower incomes no relation was found (OR = 1.00). Higher numbers of information and emotional support connections were related to more physical activity, however only for higher incomes (OR 1.20 and 1.30, respectively). Attending community organizations was positively related to physical activity, however only for patients with a low income (OR = 1.53). A healthy diet and non-smoking were less related to the social support indicators, with some exceptions. The number of household members was negatively associated with a healthy diet (OR = 0.91) and having a spouse was related to a more healthy diet in the high income group (OR = 1.67). Having more emotional support members in a network was negatively related to non-smoking (OR = 0.87). Living with more household members was positively associated with non-smoking, however only for the low income group (OR = 1.21) and attending community organizations was positively related to non-smoking, but only for higher incomes (OR = 1.72). Effects per individual country are provided in S1 File.

**Table 3:** Logistic regression estimates (OR) for the relation between social support and health-related lifestyles

	Physical activity			Healthy diet			Non-smoking		
	Overall multi-variate	Low income group	High income group	Overall multi-variate	Low income group	High income group	Overall multi-variate	Low income group	High income group
*Age (10 year steps)*	0.97	0.95	1.07	1.34**	1.41**	1.23*	1.95**	2.00**	1.91**
*Sex (male ref*.*)*	0.63**	0.59**	0.73*	1.50**	1.63**	1.29	1.44*	1.48*	1.36
*Education*	1.04**	1.06**	1.01	1.05**	1.06**	1.04*	0.99	0.96	1.03
*Non-native background*	0.79	0.94	0.79	0.69*	0.95	0.48*	0.94	1.27	0.71
*No comorbidities (ref*.*)*									
*1–2 Comorbidities*	0.71*	0.63*	0.78	0.58**	0.59**	0.52**	0.94	1.54	0.43*
*> 2 Comorbidities*	0.40**	0.39**	0.41**	0.55**	0.55**	0.54*	0.85	1.26	0.38*
*Low income*	0.75*			1.06			0.83		
**Social support**									
*Spouse*	1.07	1.09	1.19	1.05	0.90	1.67*	0.93	0.84	1.29
*Household members*	1.00	1.04	0.92	0.91*	0.90	0.92	1.10	1.21*	0.91
*Support network members (N)*	0.92	1.04	0.77**	1.03	1.06	0.99	1.14	1.17	1.15
*Network members providing*:									
*Information support*	1.09*	1.01	1.20*	1.06	1.03	1.11	1.02	1.04	0.95
*Practical support*	0.90*	1.00	0.72**	1.03	0.99	1.08	0.94	0.93	0.97
*Emotional support*	1.10*	1.01	1.30**	0.96	1.00	0.92	0.87*	0.87	0.83
*Health professional in wider network*	1.11	0.90	1.44*	1.10	1.10	1.05	0.98	0.84	1.25
*Attending community organizations*	1.18	1.53**	0.79	1.04	1.15	0.91	1.32	1.15	1.72*
*Neighborhood (urban affluent = ref*.*)*									
*Urban deprived*	0.94	0.74	1.11	0.92	0.83	1.01	0.83	0.97	0.71
*Rural deprived*	0.82	0.78	0.87	1.07	0.99	1.37	0.99	0.98	1.10

* p <0.05

** p <0.01

## Discussion

This study found that across Europe patients with a diagnoses of diabetes reported variable availability of social support from individuals and community organizations. Participation in community organizations (reported by about a third of the population) was most consistently related to better health status and health-related behaviors, especially in low income populations. Individual support network characteristics had mixed effects on health and behaviors, while living in a deprived urban neighborhood had a negative impact on mental health status. These linkages were influenced by individual income, which itself had a (positive) effect on health and health-related behaviors. In low income populations, some of the positive impacts of a large individual support network were not found.

Our findings are consistent with other studies that focus on the influences of social support and social networks. The mixed effect of individual support networks was also found in a systematic review which indicated tentative evidence for informal support [30]. The contagion of health-related behaviors (such as smoking) as found by Fowler and Christakis in the Framingham cohort may have to be reconsidered, given our finding that some of the protective impacts of a large individual support network were only found in patients with high income [31,32]. This suggests that deprived patients benefit less from a large network than those with high income. Regardless of the hypothesized mechanisms of social support, this finding raises important concerns about the potential untapped resources in individual networks to compensate for austerity measures, particularly in people with low income.

On the other hand, participation in community organizations had a consistently (small) positive effect on health status and physical activity, especially in low income groups. Besides directly providing information, practical help and emotional support, or navigating to sources of support, these organizations can fulfill a range of functions including enhancing feelings of social integration and individual identity [33]. An alternative explanation for this relation between physical health and community organizations could be that a poor physical health resulted in less community organizations visits, suggesting a different causality. However, if a good physical health allowed patients to visit a community organization, one would expect that this association was found in both the high and low income groups. Interestingly, qualitative interviews with individuals with diabetes suggested that providing support to others was one of the key mechanisms of support that contributed to better health status [34].

A strong and novel aspect of this study is that the involved multiple settings that reflect a variety of European countries. Moreover, these countries differ in health and welfare systems and policies in response to austerity in Europe. The focus on regions made it possible to combine various types of social support (from individual networks, community organizations, and neighborhoods) in one analysis. The cross-sectional design of the study did not allow causal inferences, so we could only speculate about mechanisms underlying social support. While we used previously validated measures and methods, the study has a risk of bias due to non-identified differences in national health systems and cultures.

Further research could explore how different types of social support networks differ between patient characteristics such as gender, age, and income level. More inside in the differences between groups could provide an indication of the potential to increase social support networks. Some indication is provided by a study in the UK showing that non-white and more affluent participants received slightly higher amounts of everyday work support, however effects on illness related and emotional support were not found [35]. Also it is known that women have larger and more supportive networks than men [36]. However, interventions to improve social support found mixed results; a review on social support interventions could not clarify which aspects of social support were most effective for enhancing self-management and outcomes of care for people with type 2 diabetes [37]. Therefore, more research on the development of successful interventions targeting social support is necessary and this study provides an indication for directions of new interventions.

An important implication of this study for health professionals and policy makers is that they may need to give consideration to the provision of (increased) support to community organizations, which offer activities that are relevant for the self-management of health in people with chronic diseases. Although our study was not designed to provide nationally representative samples, it also suggests that there is room to increase the participation of relevant groups in these organizations. Many diabetes patients reported receiving support from family members, friends and others. The relevance of having a large number of connections was mixed and overall limited, particularly in low-income groups. Therefore, interventions to increase the size of individual support networks need to be applied on the basis of individual assessments rather than taken as the given goals of public health policies.

## Supporting Information

S1 File Additional web tables. Regression analysis per country. (DOCX)
