# Supplementary material for: Social Support and Health in Diabetes Patients: An Observational Study in Six European Countries in an Era of Austerity
Source: PLoS One. 2015 Aug 25;10(8):e0135079. doi: 10.1371/journal.pone.0135079 (PMC4549295; doi:10.1371/journal.pone.0135079)
Supplement: S1 File — (DOCX) [file pone.0135079.s001.docx]

*Supplemental table 1. Linear regression estimates (B) for the relation between social support and physical health status per country*

|  | *Bulgaria* | *Greece* | *Nether-lands* | *Norway* | *Spain* | *UK* |
| --- | --- | --- | --- | --- | --- | --- |
| *Age (10 year steps)* | -0.47 | -0.96* | -0.38 | -0.27 | 0.12 | -0.53 |
| *Sex (male ref.)* | 0.11 | 0.03 | -2.56** | -0.69 | -1.52* | -1.42 |
| *Education* | 0.36** | 0.32** | -0.15 | 0.06 | 0.14 | -0.02 |
| *Non-native background* | -1.14 | 2.58* | -0.37 | -1.25 | -0.36 | - † |
| *No comorbidities (ref.)* |  |  |  |  |  |  |
| *1-2 Comorbidities* | -4.82** | -2.52 | -1.41 | -0.61 | -3.11** | -2.14* |
| *> 2 Comorbidities* | -7.37** | -5.75** | -3.40** | -2.15* | -6.33** | -3.87** |
| *Low income* | -1.88* | 0.65 | -1.73* | -4.00** | -2.21* | -2.34* |
| **Social support** |  |  |  |  |  |  |
| *Spouse* | 0.47 | 0.99 | 1.53 | 2.47** | 0.48 | 0.74 |
| *Household members* | -0.10 | 0.53 | 0.00 | -0.37 | -0.26 | -0.54 |
| *Support network members (N)* | -0.31 | -0.35 | 0.12 | 0.19 | -0.34 | 0.14 |
| *Network members providing:* |  |  |  |  |  |  |
| *Information support* | -0.16 | 0.60 | -0.48 | 0.02 | -0.04 | 0.18 |
| *Practical support* | -0.24 | 0.41 | -0.98** | -0.60* | -0.55 | -0.73* |
| *Emotional support* | 0.29 | 0.20 | -0.09 | 0.16 | 0.50 | 0.30 |
| *Health professional in wider network* | 1.39* | 0.23 | 1.61* | -1.29* | 0.87 | 0.26 |
| *Attending community organisations* | 0.80 | 0.29 | 1.69** | 0.31 | 2.87** | 1.22 |
| *Neighbourhood (urban affluent = ref.)* |  |  |  |  |  |  |
| *Urban deprived* | -0.45 | 1.80* | -0.85 | 0.47 | 0.90 | -2.36** |
| *Rural deprived* | -0.74 | 3.40** | -1.40 | 0.55 | 1.39 | - ‡ |

* p <0.05, ** p <0.01

‡ Not included in sampling, † not recorded.

*Supplemental table 2. Linear regression estimates (B) for the relation between social support and mental health status per country*

|  | *Bulgaria* | *Greece* | *Nether-lands* | *Norway* | *Spain* | *UK* |
| --- | --- | --- | --- | --- | --- | --- |
| *Age (10 year steps)* | 0.50 | -0.27 | 0.59 | 0.56* | 0.90** | 0.60* |
| *Sex (male ref.)* | -0.50 | -0.83 | -2.17** | -0.38 | -1.77* | -1.44* |
| *Education* | 0.32** | 0.26** | 0.07 | 0.00 | 0.13 | -0.08 |
| *Non-native background* | 2.39 | 1.55 | -1.74 | -1.19 | 0.02 | -† |
| *No comorbidities (ref.)* |  |  |  |  |  |  |
| *1-2 Comorbidities* | -4.77** | -2.16 | 0.80 | 0.67 | -1.81* | -1.55* |
| *> 2 Comorbidities* | -6.77** | -5.41** | -1.73 | -0.45 | -4.28** | -2.80** |
| *Low income* | -2.16** | -0.07 | -0.99 | -1.75** | -1.87* | -0.93 |
| **Social support** |  |  |  |  |  |  |
| *Spouse* | 0.33 | 0.89 | 0.75 | 1.10 | 0.36 | 1.38 |
| *Household members* | 0.17 | 0.26 | 0.61 | 0.01 | 0.08 | -0.41 |
| *Support network members (N)* | 0.07 | 0.34 | 0.16 | 0.22 | -0.07 | 0.09 |
| *Network members providing:* |  |  |  |  |  |  |
| *Information support* | 0.51 | 0.86* | -0.21 | 0.17 | -0.12 | 0.06 |
| *Practical support* | 0.01 | 0.16 | -0.79* | -0.09 | -0.49 | -0.39 |
| *Emotional support* | -0.78 | -0.39 | 0.07 | 0.06 | 0.18 | 0.02 |
| *Health professional in wider network* | 1.19 | 0.12 | 1.34* | 0.31 | 0.84 | 0.48 |
| *Attending community organisations* | 0.77 | 0.79 | 0.52 | 1.11 | 1.90* | 0.60 |
| *Neighbourhood (urban affluent = ref.)* |  |  |  |  |  |  |
| *Urban deprived* | -0.30 | 0.86 | -0.59 | -0.80 | -0.17 | -1.43* |
| *Rural deprived* | 0.54 | 1.43 | -1.08 | -0.45 | 0.78 | -‡ |

* p <0.05, ** p <0.01

‡ Not included in sampling, † not recorded.

*Supplemental table 3. Logistic regression estimates (OR) for the relation between social support and physical activity per country*

|  | *Bulgaria* | *Greece* | *Nether-lands* | *Norway* | *Spain* | *UK* |
| --- | --- | --- | --- | --- | --- | --- |
| *Age (10 year steps)* | 0.87 | 0.86 | 0.85 | 1.29 | 0.92 | 1.14 |
| *Sex (male ref.)* | 0.88 | 0.44* | 0.76 | 0.76 | 0.37** | 0.72 |
| *Education* | 1.11 | 1.05 | 1.00 | 1.03 | 1.06 | 0.98 |
| *Non-native background* | 0.00 | 1.20 | 0.92 | 0.56 | 2.61 | -† |
| *No comorbidities (ref.)* |  |  |  |  |  |  |
| *1-2 Comorbidities* | 0.40 | 0.32* | 1.17 | 0.85 | 0.50 | 0.96 |
| *> 2 Comorbidities* | 0.19** | 0.13** | 0.51 | 0.39* | 0.52 | 0.88 |
| *Low income* | 1.25 | 0.91 | 0.67 | 0.53* | 0.77 | 0.73 |
| **Social support** |  |  |  |  |  |  |
| *Spouse* | 1.12 | 1.06 | 1.14 | 0.90 | 0.83 | 1.90 |
| *Household members* | 0.95 | 1.17 | 0.81 | 1.14 | 1.01 | 0.77 |
| *Support network members (N)* | 2.39 | 0.77 | 1.00 | 0.94 | 0.62 | 0.86 |
| *Network members providing:* |  |  |  |  |  |  |
| *Information support* | 0.92 | 1.15 | 1.23 | 1.05 | 1.12 | 1.20 |
| *Practical support* | 1.00 | 0.76 | 0.86 | 0.82 | 0.85 | 0.99 |
| *Emotional support* | 0.52 | 1.49 | 0.91 | 1.16 | 1.56* | 1.01 |
| *Health professional in wider network* | 1.14 | 1.20 | 1.88* | 0.89 | 1.04 | 1.14 |
| *Attending community organisations* | 0.74 | 1.45 | 1.07 | 1.78 | 1.15 | 1.63 |
| *Neighbourhood (urban affluent = ref.)* |  |  |  |  |  |  |
| *Urban deprived* | 0.42 | 1.89 | 1.90 | 1.08 | 0.97 | 0.91 |
| *Rural deprived* | 1.34 | 0.35* | 1.64 | 1.11 | 0.49 | -‡ |

* p <0.05, ** p <0.01

‡ Not included in sampling, † not recorded.

*Supplemental table 4. Logistic regression estimates (OR) for the relation between social support and healthy diet per country*

|  | *Bulgaria* | *Greece* | *Nether-lands* | *Norway* | *Spain* | *UK* |
| --- | --- | --- | --- | --- | --- | --- |
| *Age (10 year steps)* | 1.27 | 0.88 | 1.16 | 1.30* | 1.96** | 1.82** |
| *Sex (male ref.)* | 0.85 | 3.72** | 1.36 | 1.28 | 1.84* | 1.02 |
| *Education* | 1.26** | 1.10* | 1.03 | 1.04 | 1.02 | 1.06 |
| *Non-native background* | 0.00 | 1.30 | 1.09 | 0.45 | 0.18 | -† |
| *No comorbidities (ref.)* |  |  |  |  |  |  |
| *1-2 Comorbidities* | 0.17** | 1.97 | 0.46 | 1.15 | 0.44 | 0.57 |
| *> 2 Comorbidities* | 0.30 | 2.36 | 1.01 | 0.61 | 0.42 | 0.35* |
| *Low income* | 2.65* | 1.01 | 0.84 | 1.02 | 1.03 | 1.38 |
| **Social support** |  |  |  |  |  |  |
| *Spouse* | 0.85 | 1.58 | 1.45 | 1.13 | 0.50 | 0.79 |
| *Household members* | 0.87 | 0.63** | 0.70 | 1.00 | 1.03 | 1.36 |
| *Support network members (N)* | 0.42 | 1.12 | 1.07 | 0.92 | 1.21 | 1.02 |
| *Network members providing:* |  |  |  |  |  |  |
| *Information support* | 1.15 | 1.00 | 1.00 | 1.29* | 0.87 | 1.12 |
| *Practical support* | 1.38 | 1.33 | 1.14 | 1.04 | 0.99 | 0.93 |
| *Emotional support* | 1.95 | 0.93 | 0.92 | 0.92 | 0.94 | 0.92 |
| *Health professional in wider network* | 1.06 | 1.09 | 1.11 | 0.68 | 1.99* | 1.66 |
| *Attending community organisations* | 0.95 | 1.44 | 0.74 | 0.85 | 1.41 | 0.84 |
| *Neighbourhood (urban affluent = ref.)* |  |  |  |  |  |  |
| *Urban deprived* | 4.06** | 0.31** | 0.57 | 0.85 | 1.17 | 1.55 |
| *Rural deprived* | 1.04 | 0.71 | 0.76 | 1.06 | 1.90 | -‡ |

* p <0.05, ** p <0.01

‡ Not included in sampling, † not recorded.

*Supplemental table 5. Logistic regression estimates (OR) for the relation between social support and non-smoking per country*

|  | *Bulgaria* | *Greece* | *Nether-lands* | *Norway* | *Spain* | *UK* |
| --- | --- | --- | --- | --- | --- | --- |
| *Age (10 year steps)* | 3.49** | 2.19** | 2.69** | 1.68** | 2.14** | 1.31 |
| *Sex (male ref.)* | 1.11 | 2.15 | 1.40 | 1.05 | 4.72** | 0.87 |
| *Education* | 0.87 | 1.08 | 1.00 | 1.04 | 1.00 | 0.93 |
| *Non-native background* | 0.00 | 1.04 | 0.51 | 1.10 | 1.00 | -† |
| *No comorbidities (ref.)* |  |  |  |  |  |  |
| *1-2 Comorbidities* | 0.90 | 0.95 | 1.48 | 0.46 | 2.91 | 1.04 |
| *> 2 Comorbidities* | 1.48 | 1.73 | 0.84 | 0.22** | 1.53 | 0.92 |
| *Low income* | 0.94 | 3.34* | 0.71 | 0.73 | 0.15* | 0.48 |
| **Social support** |  |  |  |  |  |  |
| *Spouse* | 0.51 | 1.56 | 0.30 | 1.80 | 1.12 | 0.28 |
| *Household members* | 1.32 | 0.72 | 1.03 | 1.00 | 1.07 | 2.75* |
| *Support network members (N)* | 0.91 | 1.00 | 1.47 | 0.97 | 2.16 | 0.99 |
| *Network members providing:* |  |  |  |  |  |  |
| *Information support* | 1.07 | 0.86 | 0.93 | 1.04 | 1.35 | 0.93 |
| *Practical support* | 0.59 | 0.97 | 1.48 | 1.12 | 1.00 | 0.84 |
| *Emotional support* | 1.04 | 0.00 | 0.61 | 1.05 | 0.36* | 1.05 |
| *Health professional in wider network* | 1.49 | 0.66 | 3.31* | 0.53 | 0.57 | 1.51 |
| *Attending community organisations* | 0.74 | 1.91 | 1.14 | 4.87** | 1.39 | 1.07 |
| *Neighbourhood (urban affluent = ref.)* |  |  |  |  |  |  |
| *Urban deprived* | 0.58 | 1.00 | 0.75 | 1.53 | 2.93 | 0.54 |
| *Rural deprived* | 0.51 | 0.66 | 1.16 | 1.22 | 2.16 | -‡ |

* p <0.05, ** p <0.01

‡ Not included in sampling, † not recorded.
